# Microenvironmental Reactive Oxygen Species in Colorectal Cancer: Involved Processes and Therapeutic Opportunities

**DOI:** 10.3390/cancers13205037

**Published:** 2021-10-09

**Authors:** Maria Alba Sorolla, Ivan Hidalgo, Anabel Sorolla, Robert Montal, Ona Pallisé, Antonieta Salud, Eva Parisi

**Affiliations:** 1 Research Group of Cancer Biomarkers, Biomedical Research Institute of Lleida (IRBLleida), 25198 Lleida, Spain; msorolla@irblleida.cat (M.A.S.); ihidalgo@irblleida.cat (I.H.); asorolla@irblleida.cat (A.S.); rmontal.lleida.ics@gencat.cat (R.M.); opallise.lleida.ics@gencat.cat (O.P.); masalud.lleida.ics@gencat.cat (A.S.); 2 Department of Medical Oncology, Arnau de Vilanova University Hospital (HUAV), 25198 Lleida, Spain

**Keywords:** colorectal cancer, reactive oxygen species, tumor microenvironment, antioxidants, pro-oxidants, clinical trials

## Abstract

**Simple Summary:**

Colorectal cancer is a disease associated with a high mortality rate. During the tumorigenic process, several factors and signaling molecules produced by tumor cells and the cells that surround them (forming the tumor microenvironment) regulate and modify cancer proliferation and metastasis. These regulatory agents include reactive oxygen species (ROS), which are involved in different metabolic networks and in the maintenance of cell homeostasis. Their excess, however, can cause oxidative stress and be detrimental to the cell. In fact, oxidative stress has been linked to several processes related to colorectal cancer initiation and progression. The different activities where ROS are involved suggest that ROS level modulators could be used to benefit cancer patients.

**Abstract:**

Colorectal cancer (CRC) is the fourth most common cause of cancer deaths worldwide. Although screening programs have reduced mortality rates, there is a need for research focused on finding the main factors that lead primary CRC to progress and metastasize. During tumor progression, malignant cells modify their habitat, corrupting or transforming cells of different origins and creating the tumor microenvironment (TME). Cells forming the TME like macrophages, neutrophils, and fibroblasts generate reactive oxygen species (ROS) that modify the cancer niche. The effects of ROS in cancer are very diverse: they promote cellular proliferation, epithelial-to-mesenchymal transition (EMT), evasion of cell death programs, migration, and angiogenesis. Due to the multifaceted role of ROS in cancer cell survival and function, ROS-modulating agents such as antioxidants or pro-oxidants could have therapeutic potential in cancer prevention and/or as a complement to systemic treatments. In this review, we will examine the main ROS producer cells and their effects on cancer progression and metastasis. Furthermore, we will enumerate the latest clinical trials where pro-oxidants and antioxidants have therapeutic uses in CRC.

## 1. Introduction

Colorectal cancer (CRC) is one of the most frequently diagnosed cancers and is the fourth leading cause of cancer death in both genders worldwide [1]. Screening programs have promoted early detection and a reduction in mortality rates; however, an important number of patients are still diagnosed at advanced stages [2]. Therefore, a better understanding of factors that influence tumor progression is fundamental to prevent metastasis, reduce mortality, and improve prognosis. 

Cancers are not just clonal masses of malignant cells but involve the intricate cooperation of many other cell types which are recruited and can be corrupted by the transformed ones, creating the tumor microenvironment (TME) [3]. Cells from the TME, like macrophages, neutrophils, and fibroblasts, are sources of growth factors, cytokines, proteases, and reactive oxygen species (ROS) that modify the cancer niche, acting on multiple cell types and matrix components [4]. 

ROS are endogenously generated through multiple mechanisms, with the mitochondria being their major source [5]. Excessive ROS levels cause oxidative damage to DNA, proteins, and lipids, and become detrimental to cells; therefore, the balance in ROS redox processes (which maintains physiological homeostasis) is critical, and its disruption affects several cellular processes associated with neoplastic transformation and aberrant growth [6]. The effects of ROS in cancers are very diverse: they promote cellular proliferation, evasion of apoptosis and anoikis, tissue invasion, and angiogenesis. ROS are also involved in one of the most accepted mechanisms leading to metastasis, the epithelial-to-mesenchymal transition (EMT) [7]. On the other hand, ROS play an important role in the antitumoral immune response through the activation of inflammatory and immune cells such as T lymphocytes and natural killer (NK) cells [8]. 

Herein, we present an overview of the origin and influence of ROS (with a focus on those produced by TME cells) on cancer progression and metastasis. Due to the dual role of ROS in both cell survival and cell death, there is therapeutic potential for the use of both pro-oxidants and antioxidants in CRC. In this regard, we have also reviewed the latest clinical trials where these interventions have been applied.

## 2. Oxidative Stress in Tumorigenesis

Cancer cells have high energy demands, and for this reason they require increased glycolytic activity to cover cell energy requirements and the production of anabolic intermediates. This dependence on glycolysis is independent of the presence of oxygen. Rapid glucose uptake will favor the conversion of pyruvate to lactate in the cytosol, although it is less efficient than oxidative phosphorylation in terms of ATP production is 10–100 times faster [9]. Moreover, this alternative glucose catalytic pathway will allow for the fueling of other non-mitochondrial pathways [10,11,12,13] responsible for intermediates and macromolecular synthesis, therefore ensuring tumor maintenance.

This increased glycolytic activity is known as the Warburg effect, where aerobic glycolysis predominates over mitochondrial oxidative phosphorylation [14]. Interestingly, the Warburg effect constitutes the basis for the detection of highly proliferative tumorigenic masses by measuring 2-deoxy-2-[18F]fluoro-D-glucose, a glucose analog, by positron emission tomography [15].

Cancer cells exhibit high levels of oxidative stress due to oncogenic transformations, including genetic and metabolic alterations and changes in the TME [16]. ROS are not only produced in the mitochondria but also in peroxisomes and the endoplasmic reticulum in both cancer cells and the microenvironment. The term “ROS” includes radical (superoxide, O_2_^•−^; hydroxyl, OH^•^) and non-radical (hydrogen peroxide, H_2_O_2_) oxygen-containing molecules with different reactivity [17]. Besides, reactive nitrogen species (RNS) are derived from the nitric oxide (^•^NO) produced by the activity of nitric oxide synthase (NOS). The reaction between ^•^NO and O_2_^•−^ results in the production of peroxynitrite (ONOO^−^), a highly reactive RNS that is especially harmful to proteins, particularly those containing transition metal centers [18].

Deleterious effects of ROS are counteracted by several ROS scavenging mechanisms. These are enzymatic-based systems (glutathione peroxidase, thioredoxin peroxidases, superoxide dismutases, peroxiredoxins, thioredoxins, glutaredoxins, cytochrome c oxidases, and catalases) and non-enzymatic antioxidants (coenzyme Q, ascorbic acid, tocopherol, vitamin E, and carotene) [19]. 

Oxidative stress is a master modulator of tumorigenesis, as reported in an excellent review by Gorrini et al. [20]. In fact, several cancer processes are redox-sensitive. These include cell cycle progression and proliferation, cell survival and apoptosis, energy metabolism, cell morphology, cell-to-cell adhesion, cell motility, angiogenesis, and tumor stemness [21]. Moreover, it has been widely described that oncogene activation triggers ROS accumulation in cancer cells [22,23]. However, it is still controversial as to whether ROS accumulation has oncogenic or tumor suppressive functions, as different studies have shown opposing results. The answer seems to be dependent on ROS levels, the stage of the cancer, and the reported differential effects triggered by ROS in cancer cells [7]. 

Low to moderate ROS levels act by promoting cell proliferation though the activation of survival cascades such as mitogen-activated protein kinase (MAPK)/extracellular signal-regulated kinase (ERK), phosphoinositide-3-kinase/protein kinase B (PI3K/Akt), cyclin D1 expression, and c-Jun N-terminal kinase (JNK) [24]. In addition, ROS trigger the reversible inactivation of phosphatase and tensin homolog (PTEN) [25]. Conversely, high ROS levels promote senescence [26], cell death [27,28,29], and cellular damage [30]. This cellular damage translates to oxidative modifications of proteins, lipids, and DNA. Interestingly, ROS trigger microsatellite instability through the induction of DNA damage [31,32]. 

Many mechanisms responsible for the development of CRC involve ROS. For instance, risk factors commonly associated with CRC pathogenesis, such as alcohol and smoking consumption, are inducers of ROS [33]. In addition, chronic gut inflammation is caused by a disruption of redox homeostasis. This is exemplified in inflammatory bowel disease and Cronh’s disease, where ROS production is exacerbated, leading to the activation of carcinogenesis-related genes and thus increasing the risk of CRC in these patients [34]. Moreover, the abnormal activation of KRAS, an oncogene mutated in approximately 35–45% of all CRC cases [35], impairs redox balance and is implicated in the activation of pro-oxidant programs [36]. In particular, the RAS proto-oncogene promotes superoxide production through the upregulation of NAPDH oxidase 1 (NOX1), a superoxide generating enzyme, through MAPK pathway activation [37]. 

Finally, cancer stem cells (CSCs), estimated to be tumor initiators due their self-renewal and differentiation capacities, possess a particular redox status. CSCs have low ROS levels, which could be due to their slower division rate compared to cancer cells [38]. These lower ROS levels make CSC less sensitive to ROS-generating therapies such as radiotherapy and chemotherapy, which is in turn believed to be responsible for cancer recurrence. Other authors suggest there is an upregulation of antioxidant mechanisms in CSC to overcome the deleterious effects of ROS from cancer cells and the TME [39].

## 3. ROS Generation by the Tumor Microenvironment

**Figure 1:**
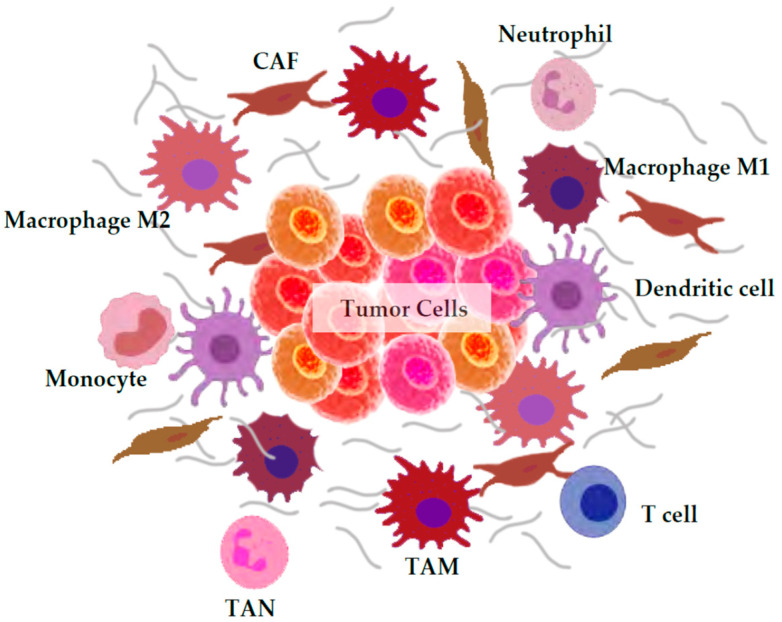
Main reactive oxygen species (ROS) producers in the tumor microenvironment (TME). The TME is a complex network of inflammatory and immune cells, fibroblasts, and stromal and epithelial cells, as well as soluble factors, signaling molecules, and extracellular matrix (ECM) components. Some of these components include ROS producers such as monocytes, macrophages (M1 and M2) and tumor-associated macrophages (TAMs), neutrophils and tumor associated neutrophils (TANs), dendritic and T cells, and cancer-associated fibroblasts (CAFs).

The biological term “TME” encompasses the entity formed by cancer cells and the wide range of non-malignant cell types and components present in the tumor, such as the immune system, components of the blood and lymphatic vascular networks, fibroblasts, the extracellular matrix (ECM), and signaling molecules [3]. The interplay between all these components and the tumor cells is essential for cancer growth, development, metastasis, and treatment response. Among all these entities, tumor cells and immune cells such as macrophages and neutrophils are major ROS producers; however, there are other minor ROS sources worth consideration (Figure 1). Depending on the type of ROS-producing cell the consequences are different and can be either tumor-supportive or tumor-suppressive [4].

### 3.1. Macrophages

The production of ROS by the immune system cells is tightly linked to the defense response and phagocytosis. It is used by neutrophils and macrophages to destroy pathogens as wells as cancer cells. Besides, these ROS are also involved in the activation of T cells and NK cells [8]. 

Macrophages constitute the major component of the infiltrate of most tumors. They can be divided into two distinct types: M1 (or classically activated) and M2 (or alternatively activated). M1 macrophages are generally considered to be tumor-killing macrophages, while M2 macrophages promote tumor growth and metastasis and are associated with poor prognosis. ROS can stimulate activation statuses in tumor-associated macrophages (TAMs) [4]. Besides, the ROS scavengers N-acetylcysteine and the NADPH oxidase ROS inhibitor diphenyleneiodonium induce monocyte polarization toward M1-like macrophages and the repolarization of M2 macrophages into M1 phenotypes. This effect prevents M2 macrophage differentiation, and, more importantly, inhibits tumor progression and M2 macrophage infiltration in the TME of CRC cell models [40].

M1 macrophages eliminate pathogens and tumor cells by secreting agents such as tumor necrosis factor α (TNF-α), interleukin (IL)-12, RNS, and ROS [41], providing a pivotal contribution in the oxidative environment. It has also been shown that monocytes activated by contact with tumor cells produce very high levels of ROS [42,43]. Moreover, TAMs, similar in phenotype to M2, are corrupted by tumor cells to promote tumor immune escape, angiogenesis, tumor growth, and metastasis [44]. The infiltration of TAMs in subcutaneous colorectal tumors is inhibited by some ROS scavengers such as Oligo-Fucoidan [40]. However, TAM-produced ROS have pro- and anti-tumorigenic activities (depending on the context), and are affected by various factors including the tumor entity and stage, as well as pre- and co-treatments [40]. 

### 3.2. Neutrophils

As in the case of macrophages, the role of neutrophils in the tumor process has been associated with defensive responses. However, some populations of neutrophils, known as tumor-associated neutrophils (TANs), could be involved in tumor growth, invasion, and angiogenesis of cancer cells, as well as in the development of metastasis [45]. Under stimulation, neutrophils generate large amounts of superoxide by activating NADPH oxidase 2 (NOX2) and hydrogen peroxide, which can modify extracellular targets and affect neighboring cell functions [46]. In this regard, it has been demonstrated that cells of myeloid origin, such as macrophages and neutrophils, can initiate tumor growth in various organs (such as the intestine) by increasing ROS production [47]. However, the effect of neutrophils on CRC tumors is not yet clear. Rao et al. showed that the intratumoral increase in neutrophils was associated with malignant phenotypes and could predict an adverse prognosis in CRC [48]. On the other hand, another study analyzed the number of neutrophils in CRC tissues and demonstrated that high levels of TANs were associated with improved overall survival in patients with stage II CRC [49]. 

### 3.3. Cancer-Associated Fibroblasts (CAFs)

The most predominant cell type in the stroma is the fibroblast, the functions of which include the renewal of ECM, the regulation of epithelial differentiation, the regulation of inflammation, and the involvement in wound healing. Activated fibroblasts such as cancer-associated fibroblasts (CAFs) secrete ROS (among other several factors) [50], which modify the environment to favor tumor development, regulate the reorganization of the connective tissue, and also facilitate metastasis through the activation of tumor neo-angiogenesis [51]. Moreover, recent studies have demonstrated that cancer cells can induce ROS overproduction in CAFs [52], contributing to a pro-oxidative TME. ROS produced by CAFs in turn enhance ROS generation in cancer cells, increasing their tumor aggressiveness [53].

### 3.4. Others

Myeloid-derived suppressor cells (MDSCs) are immature myeloid cells that play an important role in promoting tumor progression because they are involved in the immune suppression of T and NK cells [54]. In fact, one of the most important mediators of T cell suppression by MDSCs is the ROS-dependent generation of peroxynitrite [55]. T lymphocytes or T cells are another main source of ROS. Indeed, peripheral blood T lymphocytes from cancer patients have shown to have increased ROS production compared to those from healthy subjects [56]. ROS are involved in various aspects of T cell biology, including activation, differentiation, apoptosis, and antigen recognition. T cell-intrinsic ROS also influence tumor progression. Increased ROS production in T lymphocytes promote their apoptosis and tumorigenesis, influencing their immunosuppressive capacity [4]. Dendritic cells (DCs), antigen-presenting cells acting during T cell-response process, are also involved in ROS activities. ROS produced by DCs influence the anti-tumoral immune response, as it is upregulated during cross-presentation to cytotoxic T cells [57].

## 4. Consequences of Oxidative Stress in Colorectal Cancer Progression and Metastasis 

The roles of ROS in CRC initiation have classically been linked to inflammation and DNA damage [58]. However, oxidative stress is also involved in other processes related to cancer progression and metastasis, such as epithelial-to-mesenchymal transition (EMT), angiogenesis, and apoptosis. These processes are induced by molecules and regulating factors from the TME which affect tumor cell growth and the capacity to invade distant organs [59].

### 4.1. Cell Proliferation 

One of the first events in tumorigenesis is increased cell proliferation, a feature influenced by ROS signaling molecules. For instance, the expression of NOX1 in NIH 3T3 fibroblasts increases the production of superoxide anion, and at the same time causes a 10-fold elevation in hydrogen peroxide levels. This results in the expression of cell cycle and cell growth genes [60]. The fact that cancer cells produce high amounts of hydrogen peroxide [61] adds more evidence to the positive correlation between ROS levels and proliferation. In contrast, the addition of an exogenous catalase, a hydrogen peroxide scavenger enzyme, has been shown to inhibit proliferation in a dose-dependent manner in several cancer cell lines [62]. Besides, a recent preclinical study showed that the combination of traditional chemotherapy with catalases has an additive antitumoral effect in lung adenocarcinoma cells [63]. Similarly, the overexpression of glutathione peroxidase 1 (GPx1), another hydrogen peroxide detoxifying enzyme, completely suppressed tumor cell growth in nude mice bearing v-Ha-ras-transformed rat kidney epithelial cells [64]. Moreover, the enforced expression of manganese superoxide dismutase (MnSOD), a mitochondrial superoxide anion detoxifier, reduces the growth rate of the rapid-growing pancreatic human MIA PaCa-2 cell line [65]. Similar results have been observed in colon cancer cell lines, where the inhibition of NOX1 supports proliferation by modulating ROS signaling [66]. Anti-proliferative effects, induction of apoptosis, and reduction of the Warburg effect triggered by an imbalance in the redox state were observed with the addition of the flavonoid morin in CRC cell lines [67]. Furthermore, herbal melanin promotes apoptosis and inhibits the MAPK pathway in HT29 and SW620 CRC cell lines [68]. Finally, a derivative of aminobenzenesulfonamide can hamper cell proliferation and migration, inducing apoptosis in CRC cells through ROS generation [69].

### 4.2. Induction of EMT 

**Figure 2:**
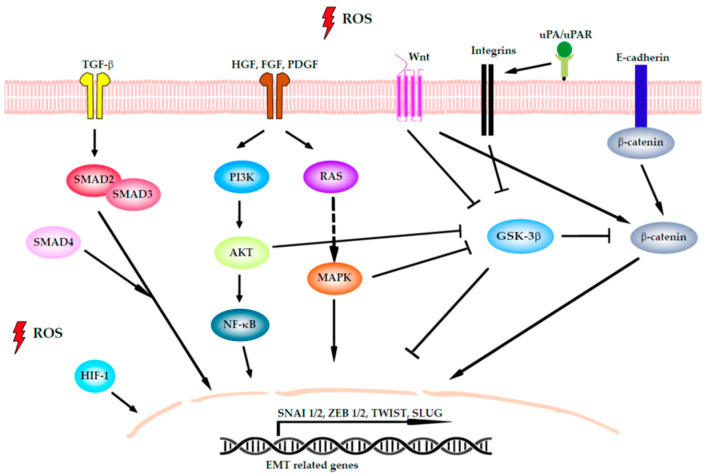
Signaling networks that regulate epithelial-to-mesenchymal transition (EMT) in colorectal cancer (CRC). The signaling pathways can induce EMT by the activation of the transcription factors (TFs) SNAI1/2, ZEB1/2, TWIST, and SLUG. Transforming growth factor β (TGF-β) induces EMT by phosphorylation of Smad2 and Smad3, which localize to the nucleus with Smad4 to activate EMT TF. Wnt inhibits glycogen synthase kinase-3 (GSK3) to stabilize β-catenin. When β-catenin is active, it translocates to the nucleus to directly activate ZEB1 and SNAI1. Several growth factors that act through tyrosine kinase receptors, such as platelet-derived growth factor growth factor (PDGF), fibroblast growth factor (FGF), and hepatocyte growth factor (HGF), promote EMT through the RAS/mitogen-activated protein kinase (MAPK) signaling cascade, the phosphatidylinositol 3-kinase (PI3K)/protein kinase B (Akt) axis, and nuclear factor-κB (NF-κB). Both pathways inhibit GSK3 as well as integrin activation, thus cooperating with Wnt signaling. The urokinase plasminogen activator (uPA) bonds to its specific cellular receptor (uPAR) to concentrate proteolytic activity at the cell surface, with this being important for extracellular matrix remodeling. Finally, the E-cadherin present on cell surface binds to cadherins on adjacent cells, whereas its intracellular region contains binding sites to interact with catenins and other regulatory proteins. When the E-cadherin/β-catenin complex is disrupted, it not only affects epithelial integrity but also the Wnt-signaling pathway. Hypoxia-inducible factor 1 (HIF-1) is activated by intracellular ROS and modulates EMT TF activity.

EMT is a well-defined process that is essential for the metastatic cascade in which tumor cells transition from an epithelial-like phenotype to a mesenchymal-like one, allowing them to escape from the basement membrane surrounding the primary tumor. During this process, cells with an epithelial phenotype lose cell–cell and cell-matrix adhesions and acquire properties of mesenchymal cells such as the ability to degrade ECM and to enhance their motility and migratory capability [70]. In CRC, as in many other cancers, the EMT process is highly regulated through some of the classic tumorigenic signaling pathways, such as the nuclear factor-κB (NF-κB), hypoxia-inducible factor 1 (HIF-1), and transforming growth factor β (TGF-β) pathways [71]. Oxidative stress plays a critical regulatory role in these pathways (Figure 2), for example by degrading their inhibitors or by inducing protein nuclear translocation and consequent transcription, as occurs with members of the NF-κB family. PI3K/Akt can facilitate protein synthesis and promote EMT, activating the NF-κB pathway. The PI3K/Akt pathway is also involved in the inhibition of glycogen synthase kinase-3 (GSK-3β), which confers stabilization to β-catenin to activate the transcript of Slug and vimentin [72]. All these inputs received by the cell lead to the activation of the EMT transcription factors (TFs) SNAI1/2, SLUG, TWIST, and ZEB1/2 [73] in colon tissues [74,75].

Aside from the role of ROS in EMT, ROS are also involved in other actions related to motility and migration. ROS mediates ECM remodeling through the arrangement of some integrins and the urokinase plasminogen activator (uPA) signaling pathways. Integrins are adhesion molecules present on the cell surface that bind the ECM with the intracellular actin cytoskeleton. The implications of ROS in regulating many integrin-mediated cellular activities are well established [76]. uPA is an extracellular serine protease for which activation by its specific receptor uPAR is required for ECM degradation and matrix metalloproteinase (MMP) activation. It has been reported that ROS induce the transcription of uPA and uPAR and stabilize their mRNA [77,78]. In this regard, Tochhawng et al. showed that gelsolin (an actin-binding protein) overexpression triggers the secretion of uPA, elevating intracellular superoxide levels in CRC cells [79]. For ECM remodeling the action of fibroblasts and CAFs in the TME is also essential. ROS levels modify CAF function by activating the TGF-β signaling pathway [80].

ROS also play an important role in actin polymerization, as they are involved in cytoskeleton remodeling and cell motility. The cytoskeleton is a dynamic network of microtubules and protein filaments where the two molecules actin and tubulin predominate and can modify their behavior under oxidation [72]. Cell motility is based on formation of actin stress fibers and actin rearrangements. Rho GTPases, which are involved in actin rearrangements, can be regulated by the focal adhesion kinase (FAK), Src, and PI3K/Akt signaling pathways, which are all modulated by ROS. Activation of the tyrosine kinase FAK leads to the recruitment of talin to nascent adhesions and the formation of focal adhesions and actin stress fibers [81]. Moreover, Src activation can enhance cell movement by promoting FC turnover and the detachment of tumor cells from the primary tumor, the latter action through downregulating E-cadherin and upregulating MMPs [82]. Besides, ROS regulate cell–cell junctions decreasing the expression of occluding, claudin, and E-cadherin, proteins repressed by the EMT master controller TF. 

### 4.3. Angiogenesis

During tumor development, new blood vessels are formed to support tumor growth. This process is called angiogenesis, and like others it is highly dependent on ROS levels [83]. Pro-angiogenic factors are activated upon physical signals such as hypoxia, ischemia, and vasculature injury. Vascular endothelial growth factor (VEGF) is of great relevance and is the primary factor initiating the angiogenic cascade, promoting the extravasation of plasma proteins and forming a primitive scaffold for migrating endothelial cells [84]. VEGF is stimulated by exogenous ROS both in vitro and in vivo [85,86]. A major endogenous ROS source in endothelial cells is from NOX activity, which could be activated by growth factors including VEGF. Interestingly, the produced ROS activate VEGF receptor 2 autophopshorylation and are involved in the activation of TFs in angiogenesis [87]. 

Each step of angiogenesis is controlled by HIF-1, which is the master regulator of oxygen homeostasis and is activated by O_2_-dependent mechanisms [88]. Under hypoxic conditions, HIF-1 upregulates many growth factors and their receptors, including VEGF and their receptors (known as VEGFRs). Additionally, activation of the PI3K/AKT/mTOR pathway in tumor cells can also increase VEGF secretion by both HIF-1 dependent and independent mechanisms. This pathway modulates the expression of other angiogenic factors such as nitric oxide and angiopoietins [89]. The expression of several mutated p53 proteins in colorectal cancer cell lines (HCT116) increases intracellular ROS and raises the number of blood vessels in their respective xenografts [90]. The crosstalk between VEGF and other oncogenes such as EGFR has been described. EGFR regulates VEGF expression via the MAPK and PI3K signaling cascades and the expression of at least three different TFs: STAT3, Sp1, and HIF-1 [91].

In CRC, HIF-1, and VEGFA are highly expressed in tumor tissue [92]. VEGFA is the isoform that has the highest affinity for VEGFR2, which is mainly found in endothelial cells. However, VEGFR1 has been found to be expressed in CRC cells, and its activation induces tumor progression and metastasis features [93]. Interestingly, intracrine VEGF signaling by CRC cells has been involved in the acquisition of cell migration and invasion phenotypes in these cells [94]. 

Increasing knowledge about the VEGF/VEGFR axis has allowed the development of novel therapeutic approaches to target angiogenesis. Initially, monoclonal antibodies against VEGF or VEGFR were proved to be effective across different treatment lines in patients with metastatic CRC. The four current anti-angiogenic drugs approved by the Food and Drug Administration are bevacizumab (anti-VEGFA), aflibercept (anti-VEGFR1), ramucirumab, (anti-VEGFR2), and regorafenib (a multikinase inhibitor including VEGFR1 and VEGFR2). Nevertheless, the lack of validated predictive markers for the different anti-angiogenic treatments is emphasized [95].

### 4.4. Apoptosis, Autophagy, and Anoikis 

The capacity to avoid cell death is one hallmark of cancer. Cell death programs, such as apoptosis, autophagy, and anoikis, serve as a natural barrier for cancer development and its activation is due to intra- and extracellular stresses that convey signals between regulators and effectors [96].

Apoptosis induced by ROS is triggered by the apoptosis signal-regulating kinase 1 (ASK1)/c-Jun N-terminal kinase (JNK) and ASK1/p38 signaling pathways in human cancer cells. When H_2_O_2_ oxidases thioredoxin 1, it dissociates from ASK1, activating the downstream MAP kinase kinase (MKK)4/MKK7/JNK and MKK3/MKK6/p38 pathways, leading to the suppression of anti-apoptotic factors [97]. ROS also activate the apoptotic pathway through death receptors and the initiator caspase 8, followed by the cleavage of downstream executor caspase 3 and Bcl-2, and finally by releasing and translocating cytochrome c [98]. Once cytochrome c is released from the mitochondria into the cytosol, it interacts with apoptotic protease-activating factor 1 (Apaf-1) to form the apoptosome, leading the activation of caspase-9 and the downstream caspase cascade [99].

In CRC, apoptosis induction via ROS in different types of cells has been demonstrated in several papers. For example, Chung et al. showed that hop proanthocyanidins or the condensed tannins found in some vegetables are cytotoxic to HT-29 colorectal adenocarcinoma cells through formation of ROS, leading to protein carbonylation and to cytoskeleton disorganization [100]. In this type of CRC cells, another set of experiments performed with resveratrol (a polyphenol found in grapes and wine) showed chemopreventive cancer properties, as this compound activated cell apoptosis through a ROS-dependent mitochondrial mechanism [101]. Resveratrol also triggered apoptosis via ROS in human CRC cells [102]. 

Autophagy is a cellular physiologic mechanism that can be strongly induced by certain cellular stresses. Cells break down cellular organelles, allowing the resulting catabolites to be used for biosynthesis and energy metabolism [96]. In this regard, it has been reported that H_2_O_2_ induces autophagic cell death in human CRC cells [103,104,105]. However, the role of the autophagy pathway in tumor progression is complex. Autophagy protects cells against the production of ROS through the elimination of the damaged mitochondria, leading to a reduction in ROS production and thereby limiting the tumor-promoting effect of ROS in DNA mutation [106].

Anoikis is a type of apoptosis induced upon cell detachment from the ECM, and is a critical mechanism for preventing adherent-independent cell growth and attachment. The deregulation of anoikis execution is an emerging hallmark of cancer cells and contributes to the formation of metastasis in distant organs [107]. ECM detachment causes a multitude of catastrophic metabolic alterations, including a robust increase in ROS [108]. It has been demonstrated that in CRC cells, anoikis can be regulated by β-catenin [109] and Src [110].

## 5. Pro-Oxidants and Antioxidants in Colorectal Cancer Therapeutics 

There is plenty of evidence that oxidative stress and ROS are genotoxic, and an excess of these is deleterious for cells. As ROS are thought to be one of the major sources of endogenous DNA damage, it can be assumed that antioxidants could be beneficial as they minimize genotoxic ROS effects, acting as chemopreventive agents [111]. On the other hand, the use of pro-oxidants to elevate ROS levels in the TME could induce malignant cell death, being beneficial and able to supplement other cancer therapies.

### 5.1. Pro-Oxidants

The most promising pro-oxidant treatments are based on ROS-dependent cancer cell death induction through the alteration of several mechanisms, including the ubiquitin-proteasome pathway, tyrosine kinase cascades, glucose metabolism, glutathione reservoir, and thioredoxin activity, etc. Herein, we discuss the traditional treatments in CRC and their association with oxidative stress. Furthermore, we examine novel ROS-dependent therapeutic approaches in clinical trials (Table 1).

#### 5.1.1. Oxaliplatin

Anti-neoplastic drugs can induce high levels of oxidative stress. This is the case for the most used and accepted treatment for advanced CRC or its metastatic form, which consists of a combination of folinic acid, 5-fluorouracil (5-FU), and oxaliplatin, namely FOLFOX6 [112]. Oxaliplatin, a platinum-based drug, in combination with 5-FU, improves its antitumor activity. Platinum-based drugs trigger a ROS generation burst, resulting in the loss of mitochondrial membrane potential [113] and a decrease in the glutathione level, leading malignant cells to die by apoptosis. Platinum is a transition metallic element able to lose electrons and positively charge ions, disturbing the normal electron flow of enzymes and substrates and ultimately affecting their catalytic activity [114]. Currently, clinical trials are focused on improving the anticancer effects of FOLFOX6 and lowering the severity of side effects with adjuvants or alternatives. 

#### 5.1.2. Arsenic Trioxide (AT)

AT is a potent oxidant which acts by reducing the intracellular redox buffering capacity and promoting apoptosis in malignant cells through JNK activation [115]. It is indicated for the treatment of a specific form of acute promyelocytic leukemia [116]. AT may sensitize CRC cells to 5-FU and leucovorin treatment. A phase I trial is studying the side-effects and best dose combination of AT with 5-FU and leucovorin in patients with stage IV colorectal cancer who have relapsed or did not respond to treatment. (NCT00449137).

#### 5.1.3. Tyrosine Kinase Inhibitors (TKIs) 

TKIs are the cornerstone treatment of many cancers. TKIs are small molecules that interfere with the autophosphorylation, dimerization, and activation of the kinase, acting as receptor antagonists. There are several TKI that differ in their pharmacological effects, side effects, and target kinases. The antitumor effects of TKI result in mitochondrial dysfunction and the uncoupling of electron transport chain proteins, increasing ROS levels [117]. Imatinib is a tyrosine kinase inhibitor which targets BCR-ABL, c-KIT, and PDGFR, and is used in a wide range of cancers [118]. The utility of imatinib as a first-line therapy in combination with XELOX and bevacizumab has been investigated in stage IV patients in a phase I/II trial (NCT00784446). Results showed tolerable toxicity and promising efficacy [119]. Erlotinib is another TKI which specifically inhibits EGFR signaling and has been found to induce metabolic oxidative stress through NOX4 activation [120]. A clinical trial is currently evaluating the efficacy and safety of erlotinib in combination with permetrexed in metastatic CRC refractory to standard chemotherapy (NCT02723578). Finally, vemurafenib, a TKI indicated for the treatment of melanoma harboring the mutation V600E in BRAF, has been shown to activate oxidative metabolism and promote ROS-dependent cell death [121]. At the clinical level, vermurafenib is under evaluation for its efficacy and safety with the administration of FOLFIRI (folinic acid, 5-FU and irinotecan) plus cetuximab in advanced CRC with the BRAF V600E mutation (NCT03727763).

#### 5.1.4. Endoplasmic Reticulum (ER) Stress Inductors 

ER stress inductors are part of the anti-cancer drug armamentarium. ER stress is a common event occurring when folding protein machinery is overloaded, resulting in the accumulation of damaged proteins in the ER. In turn, cells activate the unfolded protein response, which restores homeostasis or activates cell death [122]. Several ER stress inductors have been pharmacologically developed as a strategy to kill cancer cells. The proteasome inhibitor bortezomib binds to the active site of the subunit 20S of the proteasome, leading to the accumulation of unfolded and damaged proteins that, in its turn, induces ER stress and calcium and cytochrome C release, processes that lead to apoptosis [123]. Bortezomib has been evaluated in a phase II trial to study its effectiveness in metastatic or recurrent CRC (NCT00052507). Unfortunately, bortezomib was ineffective in controlling metastatic CRC disease, but a significant accumulation of HIF-1α was seen in tumor specimens and xenograft models, suggesting that proteasome inhibition could alter the response to tumor hypoxia [124]. Another drug that causes ER stress is celecoxib, a selective cyclooxygenase-2 inhibitor that can bind and inhibit the sarcoplasmic and the ER calcium ATPase, causing ER stress through calcium leakage into the cytosol and finally resulting in apoptosis [125]. Celecoxib is currently under investigation in a phase IV trial aiming to evaluate its anticancer effect as an adjuvant therapy with the FOLFIRI regimen in patients with metastatic CRC (NCT03645187)

#### 5.1.5. Novel Anthracyclines: AVA6000

A first-in-human study has been initiated with AVA600 for patients with locally advanced and/or metastatic solid tumors including CRC (NCT04969835). This promising phase I study will evaluate the safety, tolerability, and pharmacokinetics of AVA6000, a modified pro-drug version of doxorubicin that remains inactive until it reaches the malignant microenvironment. There, once activated as doxorubicin, it attacks malignant cells, triggering multifactorial toxicity that involves oxidative stress by induction of O_2_^•−^ and H_2_O_2_, DNA/RNA damage by binding and blocking topoisomerases, autophagy and apoptosis induction by calcium leakage and calcium channel dysregulation, and mitochondrial dysfunction through ^•^NO release [126]. 

#### 5.1.6. Poly (ADP-Ribose) Polymerase (PARP) Inhibitors 

PARPs are a family of enzymes responsible for the transfer of ADP-ribose to proteins in a reaction named ribosylation. PARPs play an important role in DNA repair, specifically in base and nucleotide excision repair, when DNA damage increases. Considering that cancer cells are usually defective in homologous recombination DNA repair pathways, it is thought that they greatly rely on PARP-mediated DNA repair for survival. PARP inhibitors (PARPi) affect DNA repair and act by provoking genomic instability and accumulation of damaged cells and consequently cell cycle arrest [127]. Some studies in ovarian cancer cell lines showed that PARP inhibition decreased their proliferation by increasing ROS levels and oxidative damage in all cancer lines analyzed [128]. At the clinical level, PARPi are the chosen treatment for certain breast and ovarian cancers, either as a single agent or in combination [129]. Two PARPi have been tested in CRC: olaparib and veliparib. Olaparib was tested in an interventional phase I trial to determine its safe dose in combination with irinotecan hydrochloride in patients with advanced or metastatic CRC (NCT00535353). Unfortunately, this combination was ineffective, dissuading researchers from further investigation [130]. Another PARPi, veliparib, was studied in combination with FOLFIRI +/− bevacizumab to evaluate its efficacy and tolerability in untreated CRC patients in a phase II trial (NCT02305758). Nevertheless, the addition of veliparib to the regimen did not provide superior efficacy [131].

### 5.2. Antioxidant Treatments or Interventions 

Whether the enhancement or inhibition of antioxidants is beneficial or detrimental for cancer treatment is still a current controversial topic. The trend in favor of the beneficial effects of antioxidants hypothesizes that their supplementation to cancer patients can be beneficial in cancer prevention as it reduces the malignancy risk or provides an additive effect for the given chemotherapy. The other line of thought, which hypothesizes that antioxidants benefit malignant cells, considers that antioxidants promote the adaptation and survival of cancer cells in the hostile microenvironment.

Herein, we show interventions based on antioxidants which are under study in CRC patients in clinical trials (Table 2).

#### 5.2.1. Dietary Supplementation of Vitamins 

A phase II trial currently recruiting patients will evaluate the effect of Ocoxin®-Viusid® on the quality of life of patients with metastatic colorectal adenocarcinoma, with the aim of enhancing tolerance to chemotherapy (NCT03559543). Ocoxin®-Viusid® is an oral solution that includes several vitamins with anticancer activity such as vitamin B6, vitamin C, and cinnamic acid, among others [132,133,134]. Similarly, a phase II/III trial intends to compare the effects of vitamin B6, folic acid, and dietary supplementation with vitamin C on homocysteine status, oxidative stress markers, antioxidant enzymatic activities, and DNA methylation in a group of 500 randomized patients with histologically confirmed CRC (NCT01426490). Other vitamin sources are tocopherols, a group of fat-soluble compounds commonly found in vegetables (α, β, γ, and δ tocopherols), many of which have vitamin E activity. Due its structure, tocopherols have unmethylated carbons which can trap ROS, acting as antioxidants. Oral uptake of vitamin E has shown to exhibit an inhibitory growth effect against malignant cancers, including CRC, in different animal models of carcinogenesis [135,136]. The use of γ-tocopherol, the major form of vitamin E in US diet, has been tested in the clinic as a pre-operatory strategy (NCT00905918). In that trial, patients with confirmed CRC scheduled for surgery received oral supplementation with γ-tocopherol. The aim was to halt the development of cancer before surgery and to study the effects of vitamin E on plasma levels of oxidative and nitrosative biomarkers. Although the trial is completed the results are not yet available.

#### 5.2.2. Trace Element Supplementation 

Trace elements are minerals present in small amounts in our organism. In that group we can find zinc, an important cofactor of nucleic acid metabolism, replication, growth, and antioxidant activity [137]. Zinc supplementation was used within cycles of chemotherapy in patients with CRC, and changes in oxidative stress during chemotherapy after surgery were assessed (NCT02106806). Results derived from this trial showed an increase in superoxide dismutase (SOD) activity attributable to zinc supplementation, with an improvement in the patient quality of life. Nevertheless, no effect of zinc complementation was seen on oxidative stress markers in plasma such as vitamin C, vitamin E, malondialdehyde, and 8-isoprostane [138].

#### 5.2.3. SOD Mimetics 

Calmangofodipir is a manganese metabolite which possesses mitochondrial MnSOD mimetic activity. A phase I/II clinical trial was designed to analyze whether the pre-treatment with calmangafodipir could decrease the frequency and severity of side effects derived from FOLFOX6 administration in patients with metastatic CRC (NCT01619423). Results from this trial demonstrated that calmangafodipir prevents the development of oxaliplatin-induced peripheral neuropathy, but no influence was observed in tumor regression [139]. Interestingly, preclinical data indicate a differential effect of this compound administered with chemotherapy between normal and malignant cells, where apoptosis is induced because of an oxidative stress burst [140,141].

#### 5.2.4. Polyphenols 

Polyphenols are a family of organic compounds abundantly found in plants. Recent polyphenol studies have shown evidence that long-term consumption of rich diets in polyphenols may protect against cancer (among other diseases) [142]. One relevant polyphenol-containing fruit is pomegranate, which has strong antioxidant effects. The active principle is ellagitannin, which is converted into urolithin A in the human gut, increasing peroxiredoxin expression which regulates peroxide levels [143]. Some human studies are focused on the effect of urolilthin A. In this regard, an interventional phase I/II trial was performed to study the effect of two pomegranate extracts in CRC patients after diagnosis until surgery. The aims of this work were to evaluate phenolic disposition and urolithins in both tumor and non-tumor colon tissues, as well as to evaluate gene expression profiling to understand the anti-inflammatory and anti-cancer effects (NCT01916239). Authors demonstrated that oral intake of pomegranate extract was significantly associated with the expression of CD44, CTNNB1, CDKN1A, EGFR, and TYMs in colon samples. However, the authors admitted that these findings were not correlated with the individual capacity to produce specific urolithins or the levels or urolithins in the colon tissues [144]. Another important polyphenol is resveratrol, found in high concentrations in red grapes. Resveratrol has shown a plethora of therapeutic benefits, including anti-inflammatory, ROS-scavenging, immunomodulatory, and anti-carcinogenic properties, among others [145]. In a phase I trial, resveratrol was given to CRC patients before surgical resection of the tumor (NCT00256334). There, researchers sought a better understanding of the effects of resveratrol in Wnt signaling pathway, which is aberrantly activated in 85% of CRCs. Surprisingly, resveratrol administration exerted inhibitory effects on Wnt signaling only in normal colonic mucosa [146]. Another subclass of polyphenol compounds is that of catechins, present in a wide variety of foods including tea, apples, persimmons, cacaos, grapes, and berries. Catechins seem to have dual action in ROS, acting as antioxidants or pro-oxidants [147]. Catechins exert their antioxidant effect by reducing free radicals through the donation of one electron of their phenolic group. Moreover, they are capable of chelating metal ions involved in radical production. Indirectly, catechins upregulate the activity of antioxidant enzymes such as superoxide dismutase, catalase, and glutathione peroxidase, and can inhibit pro-oxidant enzymes [148]. At the clinical level, catechins are under study in a phase II trial (NCT01606124) to determine whether polyphenon E green tea extract administration can prevent or delay disease progression in patients with a high risk of recurrent CRC. Recently published results concluded that polyphenon E was well tolerated but did not significantly reduce the number of aberrant rectal crypt foci, considered as a surrogate endpoint biomarker of CRC [149]. Other polyphenol family members are quercetins, mostly found in flowers, vegetables, and fruits. It has been demonstrated that flavonoids such as quercetin have stronger antioxidant effects than vitamins due to their chemical structure [150]. For instance, in vivo studies using quercetin on ascites cells in Dalton’s lymphoma-bearing mice showed downregulation of total ROS levels and protein kinase C activity, improving the apoptotic potential due to an increase in caspase 4 and 9 and promoting death receptor-mediated apoptosis [151]. Quercetin supplementation was studied as a preventive measure in individuals at medium and high risk of developing CRC (NCT00003365). Unfortunately, the results have not been yet reported. Another interesting group of polyphenols are curcumoids, used worldwide for their multiple health benefits as well as their culinary and cosmetic properties. Curcumin consumption has been found to increase the serum activity of ROS-scavenging enzymes such as superoxide dismutase, catalase, and glutathione peroxidase, and reduce lipid peroxides [152]. A pilot study with curcumin is being tested in combination with 5-FU in chemorefractory metastatic CRC patients (NCT02724202). The aim of this study is to assess clinical safety and identify the clinical response rate of the combination treatment.

#### 5.2.5. Organosulfur Compounds

Sulforaphane, another natural compound, is naturally derived from cruciferous vegetables like broccoli. Sulforaphane has been described as an antioxidant indirect molecule. In this regard, sulforaphane acts as an inducer of the Keap1/Nrf2/ARE pathway [153]. Nrf2 is a transcription factor and is considered the most important regulator of antioxidant gene expression, particularly the genes responsible for glutathione synthesis [20]. One study is currently assessing the benefit of the intake of cruciferous vegetables in volunteers scheduled for screening colonoscopy. Researchers will measure suforaphane levels in blood as well as histone deacetylase expression in tissue biopsies and peripheral blood mononuclear cells (PBMCs) (NCT01344330). Results are now under examination.

### 5.3. Future Perspectives

Recently, new emerging technologies such as drug conjugates, nanoparticles, and CRISPR technology have shown great potential for multiple applications. Targeted drugs and nanomedicine can improve precision therapy, drug delivery release, diagnosis, immunotherapy, and in vivo gene and epigene editing [154,155,156,157]. In CRC, various groups have designed nanoparticles to target cancer cell surface biomarkers such as carcinoembryonic antigen (CEA) or folate receptor-α to direct the conventional chemotherapeutic treatments to the tumor. Other approaches exploit the specific properties of the TME, such as the pH, to controllably release the content from the nanoparticle to the tumor [158]. Some of these studies have taken the first step towards clinical trials. One example is a phase I/II trial administering the C’Dot drug conjugate ELU001 (which targets the tumor-overexpressed folate receptor-α) in patients with advanced tumors including colon cancer (NCT05001282).

Several studies performed in cellular and animal models use nanoparticles containing antioxidant and pro-oxidant compounds to prevent tumor formation or promote tumor apoptotic cell death by ROS species, respectively [159,160]. 

As for CRISPR technology, genome-wide CRISPR screens have served for the identification of oxidative stress-responsive genes in CRC such as Galectin-2 (Gal2) which has a tumor-suppressive role in this cancer [161]. Moreover, House et al. engineered dCas9-Killer Red to generate oxidative stress at the desired genomic region that could be used to model more natural DNA damage [162].

Undoubtedly, there will be an increasing presence of these novel technologies in the near future to precisely tackle pro-oxidant and antioxidant genes and processes, favoring the development of more efficient and selective therapies for CRC. 

## 6. Conclusions

The involvement of oxidative stress in the tumorigenic process is still a controversial topic. ROS produced either by tumor cells or by TME cells have very diverse and sometimes opposing effects on the evolution of cancer. Low to moderate ROS levels promote cell proliferation, EMT, and angiogenesis. Conversely, high ROS levels favor apoptosis, cell death, and cellular damage. Moreover, ROS influence the immune response against tumors, as they are used by macrophages and neutrophils to destroy cancer cells and activate T and NK cells. Overall, the effects seem to depend on ROS levels, the cancer stage, and the differential outcomes seen in tumor cells because of ROS exposure. 

Such a duality of ROS effects in cancer cells and TME allows us to assume that their modulation can be exploited for cancer prevention and treatment. On the one hand, antioxidants could counteract the deleterious consequences of ROS. On the other hand, pro-oxidants could induce ROS-dependent cancer cell death. However, the results do not seem to be conclusive at this point in either case. Further research is needed to provide insights on the role of ROS modulators in the initiation and progression of CRC.

## Figures and Tables

**Table 1:** Pro-oxidant compounds administered to colorectal cancer patients in clinical trials (https://clinicaltrials.gov/ accessed on 12 August 2021).

Drug or Treatment	Mechanism of Action	Interventional Arms	Inclusion Criteria	Endpoints	Phase	Number of Subjects	Status	Results	Start Date	Identifier
**Picoplatin**	A platinum-based drug that increases intracellular ROS levels	Arm 1: FOLPI: leucovorin + 5-FU + picoplatin Arm 2: FOLFOX: leucovorin + 5-FU + oxaliplatin	Metastatic colorectal cancer	Primary: dose-limiting toxicity, maximum tolerated dose Secondary: Safety and efficacy	Phase I/II	43	Not specified	No	2007	NCT00478946
**Arsenic trioxide**	Potent oxidant and apoptosis inductor	Single Arm: Arsenic trioxide + 5-FU + leucovorin	Refractory/relapsed metastatic colorectal cancer	Primary: maximum tolerated dose and thymidylate synthase expression	Phase I	13	Completed	Yes PMID: 20950119	2007	NCT00449137
**Imatinib**	TK inhibitor of BCR-ABL	Single arm: XELOX (capecitabine + oxaliplatin) + bevacizumab + imatinib	Metastatic colorectal cancer	Primary: Dose-limiting toxicity Secondary: ORR and PFS	Phase I/II	51	Completed	Yes PMID: 23963139	2008	NCT00784446
**Erlotinib**	TK inhibitor of EGFR	Single arm: pemetrexed and erlotinib	Metastatic and refractory colorectal cancer	Primary: OS and PFS Secondary: OS, disease control rate and treatment-related adverse events	Phase II	50	Completed	Yes DOI: https://doi.org/10.1093/annonc/mdy281.114	2016	NCT02723578
**Vemurafenib**	TK inhibitor of BRAF	Single arm: FOLFIRI (leucovorin + 5-FU + irinotecan) + vemurafenib + cetuximab	BRAF V600E mutated advanced colorectal cancer	Primary: ORR Secondary: early tumor shrinkage and disease control rate	Phase II	30	Recruiting	No	2018	NCT03727763
**Bortezomib**	Proteasome inhibitor that promotes endoplasmic reticulum stress	Single arm: bortezomib	Metastatic or recurrent colorectal cancer	Primary: efficacy	Phase II	19	Completed	Yes PMID: 16061869	2003	NCT00052507
**Celecoxib**	Cyclooxygenase 2 inhibitor that causes endoplasmic reticulum stress	Arm 1: FOLFIRI. Arm 2: FOLFIRI + celecoxib	Metastatic colorectal cancer	Primary: number of patients with improved radiology	Phase IV	50	Recruiting	No	2018	NCT03645187
**AVA6000 or** **Pro-doxorubicin**	Targets topoisomerase in DNA replication and promotes apoptosis by oxidative stress	Arm 1: AVA6000 standard 3 + 3 scheme Arm 2: AVA6000 dose-expansion phase	Locally advanced and/or metastatic solid tumors including colorectal cancer	Primary: Dose limiting toxicities, maximum tolerated dose, adverse events Secondary: maximum drug concentration, elimination half-time, renal clearance, ORR, duration of response, PFS and OS	Phase I	80	Recruiting	No	2021	NCT04969835
**Olaparib**	PARP inhibitor; promotes DNA damage and oxidative stress	Single arm: olaparib + irinotecan	Locally advanced or metastatic incurable colorectal cancer	Primary: recommended phase II dose, safety, tolerability, dose-limiting toxicities and pharmacokinetic profile Secondary: efficacy and pharmacodynamic outcomes	Phase I	26	Completed	Yes PMID: 27075016	2007	NCT00535353
**Veliparib**	PARP inhibitor; promotes DNA damage and oxidative stress	Arm 1: FOLFIRI ± bevacizumab + veliparib Arm 2: FOLFIRI ± bevacizumab + placebo	Untreated metastatic colorectal cancer	Primary: PFS. Secondary: OS and ORR	Phase II	130	Completed	Yes PMID: 30531832	2018	NCT02305758

Abbreviators: EGFR: epidermal growth factor receptor; ORR: objective response rate; OS: overall survival; PARP: poly (ADP-ribose) polymerases; PFS: progression-free survival; ROS: reactive oxygen species, TK: tyrosine kinase.

**Table 2:** Interventions or supplementations diets with antioxidants in healthy volunteers or colorectal cancer patients in clinical trials (www.clinicaltrials.gov accessed on 12 August 2021).

Drug or Treatment	Mechanism of Action	Interventional Arms	Inclusion Criteria	Endpoints	Phase	Number of Subjects	Status	Results	Start Date	Identifier
Ocoxin®-Viusid® (vitamin B6, C, and cinnamic acid)	Nutritional and vitamin supplement with anticancer and antioxidant activity	Single arm: Ocoxin-Viusid®	Metastatic colorectal adenocarcinoma	Primary: quality of life, tolerance of chemotherapy, and nutritional status	Phase II	40	Recruiting	No	2018	NCT03559543
Vitamin C, B6, and folic acid	Vitamin supplement with anticancer and antioxidant activity	Control group: vitamin C. Arm 1: vitamin B6 Arm 2: folic acid Arm 3: vitamin B6 + folic acid	Confirmed colorectal cancer	Primary: measurement of oxidative stress (TBARS), antioxidant activities, and DNA methylation status	Phase II/III	300	Not specified	No	2011	NCT01426490
Vitamin E	Vitamin supplement with anticancer and antioxidant activity	Arm 1: no intervention Arm 2: high γ-tocopherol vitamin E mixture (1 week before surgery) Arm 3: high γ-tocopherol vitamin E mixture (2 week before surgery)	Pre-surgical patients with colorectal cancer	Primary: measurement of plasma and urine levels of tocopherols and prostaglandin E2; measurement of plasma levels of F2-isoprostane, C-reactive protein, 3-NT, and urinary levels of 8-OHdG; and measurement of tocopherols, cell proliferation and apoptosis indicators, β-catenin localization, RXR expression, cyclooxygenase-2, 8-OHdG, and 3-NT levels in colon tissue	Phase I	14	Completed	No	2009	NCT00905918
Zinc	Trace element cofactor of endogenous antioxidant enzymes	Experimental: zinc + chemotherapy (CRC patients) Placebo comparator: placebo + chemotherapy (CRC patients) Zinc control: zinc (healthy volunteers). Placebo control: placebo (healthy volunteers)	Stage II–IV colorectal patients	Primary: oxidative stress markers (SOD, GPx, MDA, isoprostane, vitamin C and E; Secondary: FACIT-F and CTCAE	Not applicable	55	Completed	Yes PMID: 26066525	2014	NCT02106806
Calmangafodipir	MnSOD mimetic activity	Arm 1: FOLFOX6 + calmangafodipir 2 µmol/kg Arm 2: FOLFOX6 + calmangafodipir 5 µmol/kg Arm 3: FOLFOX6 + calmangafodipir 10 µmol/kg Placebo arm: FOLFOX6 + 0.9% NaCl	Advanced metastatic colorectal cancer	Primary: number of patients with neuropathy grade 2 or higher	Phase I/II	186	Completed	Yes	186	NCT01619423
UrolithinA (pomegranate formulation)	Anti-inflammatory and anti-cancer activity	Arm 1: standard pomegranate extract formulation Arm 2: new pomegranate extract formulation-1 Arm 3: new pomegranate extract formulation-2	Pre-surgical colorectal cancer patients	Primary: measurement of phenolics and their metabolites in colon tissues, plasma, and urine samples; analysis of the gene expression profile in colon tissue Secondary: evaluation of the number of patients with adverse events, measurement of circulating IGF-1 and CEA levels and microRNA expression in tumoral and colon tissues	Phase I/II	60	Completed	Yes PMID: 28183047	2013	NCT01916239
Resveratrol (grape extract)	It is suggested that modulates Wnt signaling, with anti-oxidant and pro-apoptotic effects	Single arm: resveratrol	Pre-surgical colorectal cancer patients	Primary: evaluation of the modulation of Wnt signalling in vivo in colon cancer and normal colonic mucosa	Phase I	11	Completed	Yes PMID: 21188121	2005	NCT00256334
Polyphenon E (green tea catechin extract)	Reduces free radicals, chelates metal ions, upregulates antioxidant enzymes, and inhibits prooxidant enzymes	Arm I: polyphenon Arm 2: placebo	Stage I–III high-risk colorectal cancer patients	Primary: measurement of the percent change in rectal ACF before and after the intervention Secondary: study of the tolerability dose of catechin extract	Phase II	39	Terminated	Yes PMID: 33648940	2012	NCT01606124
Sulindac + rutin + quercetin + curcumin	Antioxidant effect	Arm 1: control diet Arm 2: control diet + sulindac Arms 3–5: control diet + rutin Arms 5–7: control diet + quercetin Arm 8–10: control diet + curcumin	Individuals with average or above risk for development of colon cancer	To determine and compare the response of colon epithelium to different dietary treatments and to sulindac, and to identify the lowest optimal dose of the dietary supplementation to modulate biomarkers of colon epithelial	Not applicable	130	Terminated	No	2004	NCT00003365
Curcumin	Antioxidant activity	Single arm: curcumin + 5-FU	5-FU resistant metastatic colon cancer		Early phase I	13	Active	No	2016	NCT02724202
Sulforaphane (cruciferous vegetables)	Nrf2 enhancer which promotes antioxidant gene expression	Single arm: cruciferous vegetable intake	Patients scheduled for a screening colonoscopy	Primary: to determine the correlation of sulforaphane and indole-3-carbinol urinary levels with cruciferous intake Secondary: p21 and acetylated histone expression, and HDAC activity in PBMCs and colon tissue	Not applicable	108	Completed	No	2011	NCT01344330

Abbreviators: 3-NT: nitrotyrosine; 8-OHdG: 8-hydroxy-2’-deoxyguanosine; ACF: aberrant crypt foci; CEA: carcinoembriogenic antigen; CTCAE: common terminology criteria for adverse events; FACIT-F: functional assessment of cancer therapy-fatigue; GPx: glutathione peroxidase; HDAC: histone deacetylase; IGF-1: insulin like growth factor-1; MDA: malondialdehyde; PMBC: peripheral blood mononuclear cell; RXR: retinoic acid receptor; SOD: superoxide dismutase; TBARS: thiobarbituric acid reactive substances.
